# Prevalence of Fosfomycin Resistance and Mutations in *murA, glpT*, and *uhpT* in Methicillin-Resistant *Staphylococcus aureus* Strains Isolated from Blood and Cerebrospinal Fluid Samples

**DOI:** 10.3389/fmicb.2015.01544

**Published:** 2016-01-11

**Authors:** Zhuyingjie Fu, Ying Ma, Chunhui Chen, Yan Guo, Fupin Hu, Yang Liu, Xiaogang Xu, Minggui Wang

**Affiliations:** ^1^Institute of Antibiotics, Huashan Hospital, Fudan UniversityShanghai, China; ^2^Key Laboratory of Clinical Pharmacology of Antibiotics, Ministry of HealthShanghai, China; ^3^Infection Control Unit, Huashan Hospital, Fudan UniversityShanghai, China

**Keywords:** fosfomycin, resistance, mutations, *Staphylococcus aureus*, MRSA

## Abstract

In China, fosfomycin alone or in combination with other antibiotics is commonly used in the treatment of infections caused by Gram-positive bacteria, including methicillin-resistant *Staphylococcus aureus* (MRSA). Although fosfomycin-resistant *S. aureus* strains have continued to emerge and increase, the research on them is rare. In order to determine the prevalence and mechanisms of fosfomycin resistance in MRSA clinical isolates, a total of 96 non-duplicate MRSA isolates were collected from blood and cerebrospinal fluid samples at Huashan Hospital in Shanghai, China between 2004 and 2014. Antimicrobial susceptibility testing was performed by agar dilution. Meanwhile, the fosfomycin-resistance-related genes, *fosB, murA, glpT*, and *uhpT*, were amplified by PCR and subjected to sequencing analysis. Multilocus sequence typing (MLST) was conducted to assess strain types. The minimum inhibitory concentration (MIC) of fosfomycin for the 96 MRSA strains ranged from 1.0 to >1,024 mg/L, and approximately 70% (67/96) of the isolates were resistant to fosfomycin (MIC ≥ 64.0 mg/L). Nine isolates with MICs ≥ 128 mg/L carried *fosB* gene. Twenty-five distinct mutations were detected in the *murA* (7), *glpT* (10), and *uhpT* (8) genes. While five of the *murA* mutations and five of the *glpT* mutations were observed only in fosfomycin-sensitive isolates and one of the *murA* mutation was found both in fosfomycin-resistant and fosfomycin-sensitive isolates, the remaining 14 mutations (1 *murA*, 5 *glpT*, and all *uhpT* mutations) were present only in fosfomycin-resistant isolates. MLST analysis demonstrated that the majority (46/67) of the *glpT* and/or *uhpT* mutants belong to ST5, the predominant sequence type among the fosfomycin-resistant MRSA isolates. In conclusion, there is a high rate of fosfomycin resistance in MRSA strains. The mutations in the *murA, glpT*, and *uhpT* genes are common in fosfomycin-resistant MRSA strains, and may play a greater role in the development of fosfomycin resistance than the presence of the *fosB* gene in these organisms.

## Introduction

Methicillin-resistant *Staphylococcus aureus* (MRSA) is one of the most common nosocomial pathogens, and resistant to most β-lactam antibiotics and even new antimicrobials, which has compelled clinicians to resort to some “old” antimicrobial agents (DeLeo and Chambers, 2009). Fosfomycin, which was first discovered in *Streptomyces* sp. in 1969, possesses broad-spectrum activity against both Gram-positive and Gram-negative bacteria by inhibiting bacterial cell wall synthesis (Michalopoulos et al., 2011). Specifically, fosfomycin deactivates the enzyme UDP-*N*-acetylglucosamine-3-enolpyruvyltransferase, also known as MurA (encoded by the *murA* gene), thereby irreversibly interfering with the first committed step of peptidoglycan biosynthesis (Michalopoulos et al., 2011). In recent years, the administration of fosfomycin alone or in combination with other antibiotics has been prescribed for treatment of MRSA (Falagas et al., 2009; Del Río et al., 2014; Sultan et al., 2015). However, fosfomycin-resistant *S. aureus* strains have continued to emerge and increase (Etienne et al., 1991). In China, the fosfomycin resistance rate in MRSA was as high as 29.5% in 2010 (Guo et al., 2013).

Bacterial fosfomycin resistance is attributed both to the acquisition of chromosomal mutations and to the expression of plasmid-encoded fosfomycin-modifying enzymes. Mutations in *murA* have been shown to reduce the affinity of fosfomycin for MurA (Jiang et al., 2011). Additionally, fosfomycin intake can be reduced in the presence of mutations in *glpT* and/or *uhpT*, which encode fosfomycin transport systems of bacteria (Takahata et al., 2010; Michalopoulos et al., 2011). Lastly, fosfomycin activity can be inhibited via the catalytic activity of FosA, FosB, FosC, and FosX, respectively (García et al., 1995; Fillgrove et al., 2007; Lee et al., 2012). Meanwhile, of the known plasmid-mediated fosfomycin resistance genes, only *fosB* has been detected in *Staphylococcus* sp. To date, however, most researches have focused on fosfomycin-resistant Gram-negative bacteria, and relatively few studies have been conducted in fosfomycin-resistant Gram-positive bacteria (Michalopoulos et al., 2011). The purpose of this study was to investigate the prevalence fosfomycin resistance and mutations in the *murA, glpT*, and *uhpT* genes in fosfomycin-resistant MRSA strains isolated in China.

## Materials and Methods

### Bacterial Strains

A total of 96 MRSA clinical strains were isolated from blood or cerebrospinal fluid specimens at Huashan hospital, a teaching hospital in Shanghai, China, between 2004 and 2014. *S. aureus* ATCC 29213 (American Type Tissue Culture Collection, Manassas, VA, USA) was used as a quality control strain in antimicrobial susceptibility testing experiments.

### Antimicrobial Susceptibility Testing

The minimum inhibitory concentration (MIC) of fosfomycin for each clinical strain was determined by agar dilution supplemented with Glucose-6-phosphate (25 mg/L), according to the recommendations of the Clinical and Laboratory Standards Institute (Clinical and Laboratory Standards Institute [CLSI], 2014), and results were interpreted according to European committee on antimicrobial susceptibility testing criteria (European Committee on Antimicrobial Susceptibility Testing [EUCAST], 2012) (susceptible, ≤32 mg/L; resistant, ≥64 mg/L).

### PCR Amplification

DNA of 96 MRSA strains was harvested using TIANamp bacteria DNA Kit (Tiangen, Beijing, China). The presence of the *fosA, fosB*, and *fosC*, was detected by PCR as described previously (Chen et al., 2014). *murA, glpT* and *uhpT* genes are amplified by PCR using primers listed in **Table 1**. The PCR products were sequenced to screen for mutations.

**Table 1:** PCR primers of *fosA, fosB, fosC, murA, glpT* and *uhpT* gene.

Primers	Gene	Primer sequences (5′ > 3′)	Product size	Reference
fosA-F fosA-R	*fosA*	GCTGCACGCCCGCTGGAATA CGACGCCCCCTCGCTTTTGT	217 bp	Chen et al., 2014
fosB-F fosB-R	*fosB*	CAGAGATATTTTAGGGGCTGACA CTCAATCTATCTTCTAAACTTCCTG	312 bp	Chen et al., 2014
fosC-F fosC-R	*fosC*	GGGTTACATGCCCTTGCTCA AACCCGCACAACGACAGATG	354 bp	Chen et al., 2014
murA-F murA-R	*murA*	GCCCTTGAAAGAATGGTTCGT GTTACAATACTCGACGCAGGT	1600 bp^∗^	NC_002745.2^∗∗^
glpT-F glpT-R	*glpT*	TGAATAAAACAGCAGGGCAA CACAGCTAGTATGTATAACGAC	1699bp^∗^	NC_002745.2^∗∗^
uhpT-F uhpT-R	*uhpT*	TGTGTTTATGTTCAGTATTTTGGA TCTTTCATCTCTTCACGCAC	1571 bp^∗^	NC_002745.2^∗∗^

*^∗^PCR product including surrounding sequences adjacent to target gene.*

*^∗∗^GenBank-EMBL-DDBL accession number.*

### Multilocus Sequence Typing (MLST)

*Staphylococcus aureus* sequence types (STs) were determined by comparing the sequences of the housekeeping genes *arcC, aroE, glp, gmk, pta, tpi*, and *yqiL*, as described previously (Enright and Spratt, 1999), with those in the MLST database available at http://saureus.mlst.net/ (Aanensen and Spratt, 2005).

### Nucleotide Sequence Accession Numbers

The sequences for the TypeA*_murA_*, TypeI*_murA_*, TypeII*_murA_*, TypeIII–VI*_murA_*, TypeA–E*_glpT_*, TypeI*_glpT_*, TypeII*_glpT_*, TypeIII*_glpT_*, TypeVI*_glpT_*, TypeV*_glpT_*, and TypeA–H*_uhpT_* strains have been deposited in GenBank with the following accession numbers: KT372186, KT372187, KT372185, KT372188–KT372191, KT372192–KT372196, KT372197, KT372198, KT372201, KT372199, KT372200, and KT372202–KT372209, respectively.

## Results

### Fosfomycin Susceptibility

The MICs of fosfomycin for the MRSA strains ranged from 1.0 mg/L to >1,024 mg/L. Furthermore, MIC_50_ and MIC_90_ values of these strains were 1,024 mg/L and >1,024 mg/L, respectively. According to the EUCAST criteria, 67 out of the 96 examined MRSA isolates were characterized as fosfomycin-resistant (MIC ≥ 64 mg/L).

### Prevalence of Fosfomycin Resistance Genes

Of the 96 MRSA isolates, 9 fosfomycin-resistant strains with MIC ≥ 128 mg/L contained *fosB* (**Table 2** and Supplementary Table S1), and no isolates were positive for *fosA* and *fosC*.

**Table 2:** Characteristics of 96 methicillin-resistant *Staphylococcus aureus* isolates.

MLST types	Strain No.	*fosB* gene	Mutation in *glpT* alone		Mutation in *uhpT* alone		Mutation in both *glpT* and *uhpT*		No mutation in *glpT* and *uhpT*	
			No.	Fosfomycin MIC range(mg/L)	No.	Fosfomycin MIC range(mg/L)	No.	Fosfomycin MIC range(mg/L)	No.	Fosfomycin MIC range(mg/L)
ST5	40	Negative	4	64 ~ > 1024	6	128 ~ >1024	30	1024 ~ >1024	0	–
ST239	34	Negative	2	128	2	128 ~ >1024	5	1024 ~>1024	25	1 ~ 256
ST6	1	Negative	0	-	0	-	0	-	1	1
ST30	1	Negative	1	2	0	-	0	-	0	–
ST59	4	Negative	4	1	0	-	0	-	0	–
ST88	1	Negative	0	-	0	-	0	-	1	1
ST121	1	Negative	1	2	0	–	0	-	0	–
ST398	1	Negative	1	1	0	-	0	-	0	–
ST764	3	Negative	0	-	3	256 ~ 512	0	-	0	–
ST863	1	Negative	0	-	0	-	0	-	1	32
ST5	6	Positive	1	512	2	>1024	3	>1024	0	–
ST239	1	Positive	0	-	0	-	0	-	1	128
ST764	1	Positive	0	-	0	-	1	>1024	0	–
ST2590	1	Positive	1	>1024	0	-	0	-	0	–

### Mutations in the *murA* Gene

**FIGURE 1:**
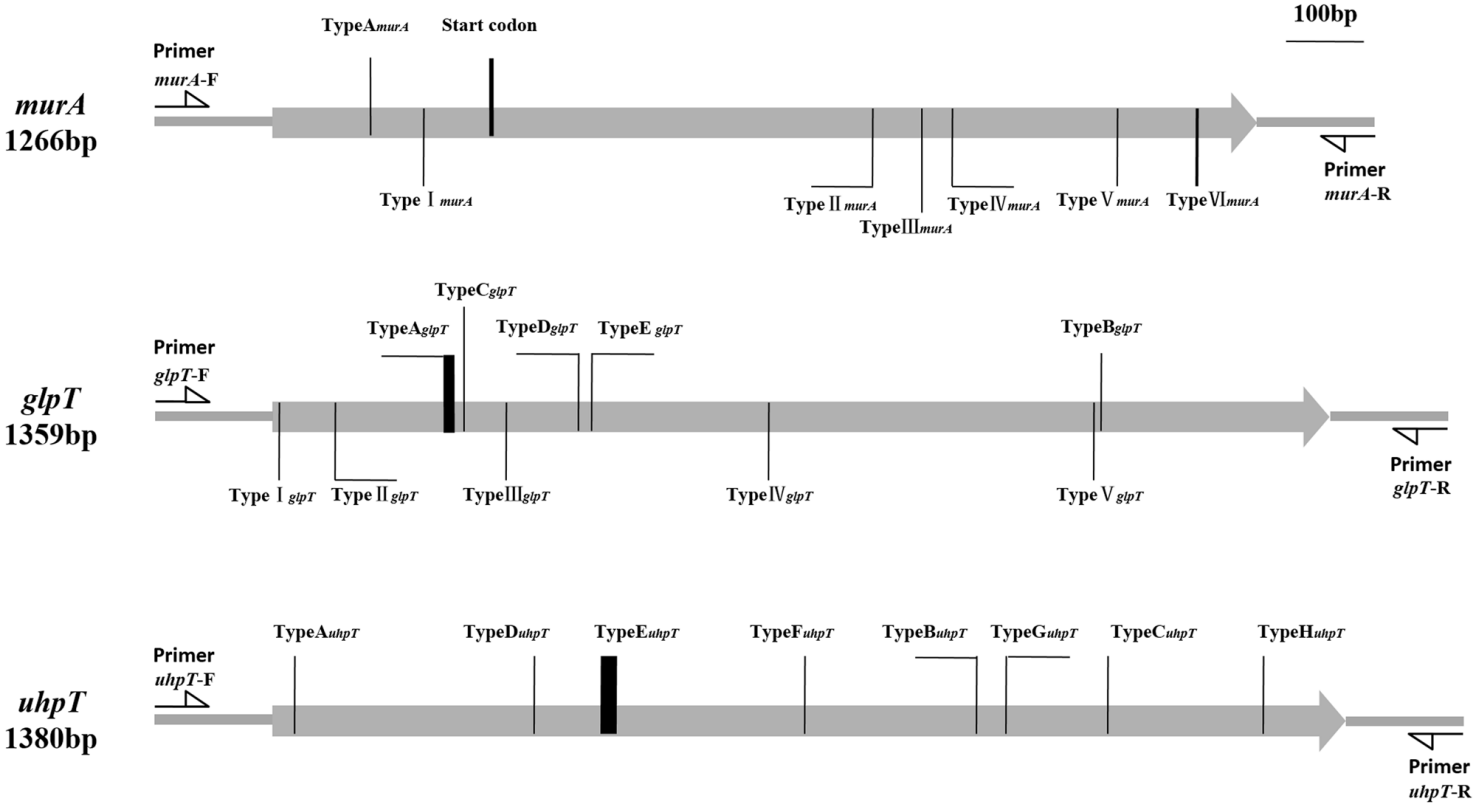
**Types and positions of mutation in *murA, glpT*, and *uhpT* genes.** TypeA*_murA_* (KT372186): T125A (Result in a possible start codon in position 94th aa); TypeI*_murA_* (KT372187): G193C; TypeII*_murA_* (KT372185): G770A; TypeIII*_murA_* (KT372188): C834A; TypeIV*_murA_* (KT372189): A873T; TypeV*_murA_* (KT372190): A1085G; TypeVI*_murA_* (KT372191): CG1187-1188AT. TypeA*_glpT_* (KT372192): Deletion of 8bp from 225T to 232A; TypeB*_glpT_* (KT372193): G1064A; TypeC *_glpT_* (KT372194): Deletion of 248G; TypeD *_glpT_* (KT372195): Insertion of 392T; TypeE *_glpT_* (KT372196): T409C; TypeI*_glpT_* (KT372197): T7A; TypeII*_glpT_* (KT372198): C79T; TypeIII*_glpT_* (KT372201): C299T; TypeIV*_glpT_* (KT372199): G637A; TypeV*_glpT_* (KT372200): G1055A. TypeA*_uhpT_* (KT372202): Deletion of 27T; TypeB*_uhpT_* (KT372203): Insertion of 904T; TypeC*_uhpT_* (KT372204): G1073T; TypeD*_uhpT_* (KT372205): G335A; TypeE*_uhpT_* (KT372206): Deletion of 12 bp from 431A to 442T; TypeF*_uhpT_* (KT372207): G683A; TypeG*_uhpT_* (KT372208): C942A; TypeH*_uhpT_* (KT372209): T1273C.

Seven distinct mutations were detected in the *murA* gene of the 96 MRSA isolates. Mutation TypeA*_murA_*, which resulted in the creation of a stop codon (TA_125_A) at position 42 and a possible new start codon was found at position 94 (**Figure 1**), was contained by one of the fosfomycin-resistant MRSA isolates. In contrast, the other six mutations (TypeI–VI*_murA_*), which resulted in distinct amino acid substitutions within the MurA protein, could be found in fosfomycin-sensitive MRSA isolates, though one mutation (TypeII*_murA_*) also could be found in fosfomycin-resistant MRSA isolates. (**Table 3**).

**Table 3:** Characteristics of MRSA isolates contained *murA* mutations.

Types of mutation	Strain No.	Mutations in *murA^∗^*	Fosfomycin MIC range (mg/L)	*fosB* gene
TypeA*_murA_*	1	T125A (Truncated to 41 aa)	1024	Negative
TypeI*_murA_*	1	G193C (Val 65 Leu)	2	Negative
TypeII*_murA_*	29	G770A (Gly 257 Asp)	2 ~ >1024	Negative
TypeIII*_murA_*	2	C834A (Asp 278 Glu)	1 ~ 2	Negative
TypeIV*_murA_*	7	A873T (Glu 291 Asp)	1 ~ 2	Negative
TypeV*_murA_*	1	A1085G (Gln 362 Arg)	2	Negative
TypeVI*_murA_*	5	CG1187-1188AT (Thr 396 Asn)	1 ~ 2	Negative

*^∗^Deduced amino acid substitutions or sequence variations are shown in brackets.*

### Mutations in *glpT* and *uhpT* Genes

Of the 67 fosfomycin-resistant MRSA isolates, 46 contained one of the five different mutations (TypeA–E*_glpT_*) observed in the *glpT* gene. While TypeE*_glpT_* resulted in an amino acid substitution at position 137 (Trp-137→Arg) of GlpT, the other four mutations produced premature stop codons within the *glpT* coding sequence, thereby resulting in the generation of truncated proteins (**Figure 1**). In addition, we detected five other mutations within the *glpT* gene that were present only in the fosfomycin-sensitive MRSA isolates. Each of these mutations (TypeI–V*_glpT_*) resulted in amino acid substitutions within the GlpT protein (Supplementary Table S2 and **Table 4**).

**Table 4:** Characteristics of fosfomycin-resistant MRSA isolates contained *glpT* and *uhpT* mutations.

Mutation and its Combination	Strain No.	ST5		ST239		Other ST types	
		No.	Fosfomycin MIC range (mg/L)	No.	Fosfomycin MIC range (mg/L)	No.	Fosfomycin MIC range (mg/L)
TypeA*_glpT_*	5	2	1024 ~ >1024	2	128	1	>1024
TypeA*_glpT_* and TypeA*_uhpT_*	1	0	–	0	–	1	>1024
TypeA*_glpT_* and TypeB*_uhpT_*	26	25	>1024	1	1024	0	–
TypeA*_glpT_* and TypeE*_uhpT_*	1	0	–	1	1024	0	–
TypeB*_glpT_*	2	2	64	0	–	0	–
TypeB*_glpT_* and TypeB*_uhpT_*	1	1	>1024	0	–	0	–
TypeB*_glpT_* and TypeC*_uhpT_*	5	5	1024	0	–	0	–
TypeB*_glpT_* and TypeF*_uhpT_*	1	1	>1024	0	–	0	–
TypeC*_glpT_*	1	1	512	0	–	0	–
TypeD*_glpT_* and TypeH*_uhpT_*	1	0	–	1	>1024	0	–
TypeE*_glpT_* and TypeC*_uhpT_*	1	0	–	1	>1024	0	–
TypeE*_glpT_* and TypeG*_uhpT_*	1	0	–	1	1024	0	–
TypeA*_uhpT_*	7	3	>1024	1	128	3	256 ~ 512
TypeB*_uhpT_*	5	5	128 ~ >1024	0	–	0	–
TypeC*_uhpT_*	1	1	1024	0	–	0	–
TypeD*_uhpT_*	1	0	–	1	>1024	0	–

Eight distinct mutations were detected in the *uhpT* gene (TypeA–H*_uhpT_*) of the fosfomycin-resistant MRSA isolates. Conversely, no *uhpT* mutations were detected in the fosfomycin-sensitive isolates (Supplementary Table S3 and **Table 4**). The TypeC*_uhpT_*, TypeD*_uhpT_*, and TypeH*_uhpT_* mutations led to amino acid substitutions within the UhpT protein, while each of the remaining mutations created premature stop codons within the *uhpT* gene, resulting in the production of truncated protein (**Figure 1**).

### Molecular Typing

The 96 MRSA isolates were categorized into 11 ST types, including ST5, ST6, ST30, ST59, ST88, ST121, ST239, ST398, ST764, ST863, and ST2590 (**Table 2**). Notably, all ST5 strains and 45.7% (16/35) of the ST239 isolates were resistant to fosfomycin (Supplementary Table S1 and **Table 2**).

## Discussion

The fosfomycin resistance rate of the MRSA isolates examined in this study was approximately 70% (67/96), which was higher than previously detected (Falagas et al., 2010). In consideration of the average fosfomycin use was as high as 300,977 DDDs (defined daily doses) per year in Shanghai hospitals between 2009 and 2014 (Unpublished data), this elevated resistance rate may have been due to the common usage of this antimicrobial in the region. However, while Etienne et al. (1991) previously detected the *fosB* gene in 46% (18/39) of fosfomycin-resistant *S. aureus* isolates, only 13.4% (9/67) of the strains examined in this study harbored the *fosB* gene. Although this gene was detected only in fosfomycin-resistant isolates, the majority of such isolates were *fosB*-negative, indicating that other mechanisms contribute to fosfomycin resistance in *S. aureus*.

Sequencing analyses detected 25 distinct mutations in the *murA, glpT*, and *uhpT* genes of the MRSA isolates tested (**Figure 1** and Supplementary Table S1). Of the seven mutations present in *murA*, 6 (TypeI–VI*_murA_*) were contained by fosfomycin-sensitive isolates, indicating that these modifications did not confer fosfomycin resistance. Also, while the TypeA*_murA_* mutation was only detected in a single fosfomycin-resistant isolate, this strain also harbored mutations within *glpT* and *uhpT*. Thus, it is unclear what role, if any, the TypeA*_murA_* mutation plays in fosfomycin resistance in MRSA.

Each of the *uhpT* mutations (TypeA–H*_uhpT_*) and five of the *glpT* (TypeA–E*_glpT_*) mutations were present only in fosfomycin-resistant isolates. Indeed, approximately 90% (60/67) of the fosfomycin-resistant strains harbored a mutation in *glpT* and/or *uhpT*. Moreover, strains that contained mutations in both *glpT* and *uhpT* exhibited high levels of fosfomycin resistance (MIC > 1,024 mg/L; **Table 2**), indicating that each of these 13 mutations might contribute to fosfomycin resistance. In contrast, the remaining five *glpT* mutations (TypeI–V*_glpT_*), which were harbored only by fosfomycin-sensitive strains, likely do not contribute to fosfomycin resistance. Notably, several fosfomycin-resistant isolates were *fosB*-negative, and did not harbor mutations in *murA, glpT*, or *uhpT*. As such, the mechanism that governs fosfomycin resistance in these isolates requires further study.

Multilocus sequence typing analyses indicated that all of the ST5 and 45.7% of the ST239 isolates were resistant to fosfomycin. The majority of the *glpT* and/or *uhpT* mutants were typed as ST5 (**Table 2**), and several strains harbored identical mutations (**Table 4**), indicating that clonal dissemination may exist. The first ST5 strain emerged in 2004 (Supplementary Table S1). The spread of this clone may be associated with the increased occurrence of fosfomycin resistant MRSA. However, fosfomycin resistance-associated mutations were also present in isolates within other STs, suggesting that the emergence of fosfomycin resistance is due to not only clonal dissemination, but also fosfomycin selective pressure on isolates from different clones, and the emergence of spontaneous mutations.

## Conclusion

We observed a high rate of fosfomycin resistance in MRSA strains isolated from Shanghai, China. Our findings indicate that mutations within the *glpT* and/or *uhpT* genes play a critical role in conferring this resistance. Thus, the continuous monitoring of fosfomycin susceptibility in *S. aureus*, particularly in MRSA strains is important. Furthermore, our results indicate that caution should be used when employing fosfomycin for the empiric treatment of MRSA infections.

## Author Contributions

Designed and conceived the experiments: YL, XX, and MW. Performed the experiments: ZF, YM, YG, and CC. Analyzed the data: FH, XX, and ZF. Wrote and reviewed the manuscript: ZF, YM, CC, YL, and XX.

## Conflict of Interest Statement

The authors declare that the research was conducted in the absence of any commercial or financial relationships that could be construed as a potential conflict of interest.

## References

[B1] Aanensen D. M. Spratt B. G. (2005). The multilocus sequence typing network, mlst.net. *Nucleic Acids Res.* 33 W728–W733. 10.1093/nar/gki415 15980573 PMC1160176

[B2] Chen C. Xu X. Qu T. Yu Y. Ying C. Liu Q. (2014). Prevalence of the fosfomycin-resistance determinant, fosB3, in *Enterococcus faecium* clinical isolates from China. *J. Med. Microbiol.* 63 1484–1489. 10.1099/jmm.0.077701-0 25102907

[B3] Clinical and Laboratory Standards Institute [CLSI]. (2014). *Performance Standards for Antimicrobial Susceptibility Testing: Twenty Fourth Informational Supplement, M100-S24* Vol. 34 Wayne, PA: Clinical and Laboratory Standards Institute

[B4] DeLeo F. R. Chambers H. F. (2009). Reemergence of antibiotic-resistant *Staphylococcus aureus* in the genomics era. *J. Clin. Invest.* 119 2464–2474. 10.1172/JCI38226 19729844 PMC2735934

[B5] Del Río A. Gasch O. Moreno A. Peña C. Cuquet J. Soy D. (2014). Efficacy and safety of fosfomycin plus imipenem as rescue therapy for complicated bacteremia and endocarditis due to methicillin-resistant *Staphylococcus aureus*, A multicenter clinical trial. *Clin. Infect. Dis.* 59 1105–1112. 10.1093/cid/ciu580 25048851

[B6] Enright M. C. Spratt B. G. (1999). Multilocus sequence typing. *Trends Microbiol.* 7 482–487. 10.1016/S0966-842X(99)01609-1 10603483

[B7] Etienne J. Gerbaud G. Fleurette J. Courvalin P. (1991). Characterization of staphylococcal plasmids hybridizing with the fosfomycin resistance gene *fosB*. *FEMS Microbiol. Lett.* 68 119–122. 10.1111/j.1574-6968.1991.tb04580.x 1769548

[B8] European Committee on Antimicrobial Susceptibility Testing [EUCAST] (2012). Available at: http://www.eucast.org

[B9] Falagas M. Maraki S. Karageorgopoulos D. E. Kastoris A. C. Kapaskelis A. Samonis G. (2010). Antimicrobial susceptibility of Gram-positive non-urinary isolates to fosfomycin. *Int. J. Antimicrob. Agents* 35 497–499. 10.1016/j.ijantimicag.2010.01.010 20226634

[B10] Falagas M. Roussos N. Gkegkes I. Rafailidis P. I. Karageorgopoulos D. E. (2009). Fosfomycin for the treatment of infections caused by Gram-positive cocci with advanced antimicrobial drug resistance, a review of microbiological, animal and clinical studies. *Exp. Opin. Investig. Drugs* 8 921–944. 10.1517/13543780902967624 19548851

[B11] Fillgrove K. Pakhomova S. Schaab M. R. Newcomer M. E. Armstrong R. N. (2007). Structure and mechanism of the genomically encoded fosfomycin resistance protein, fosx, from *Listeria monocytogenes*. *Biochemistry* 46 8110–8120. 17567049 10.1021/bi700625p

[B12] García P. Arca P. Evaristo S. J. (1995). Product of *fosC*, a gene from *Pseudomonas syringae*, mediates fosfomycin resistance by using ATP as cosubstrate. *Antimicrob. Agents Chemother.* 39 1569–1573. 10.1128/AAC.39.7.1569 7492106 PMC162783

[B13] Guo Y. Zhu D. Hu F. Wang F. Yu Y. Yang Q. (2013). CHINET 2010 surveillance and analysis of antibiotic resistance in *Staphylococcus* spp. in China. *Chin. J. Infect. Chemother.* 13 86–92.

[B14] Jiang S. Gilpin M. E. Attia M. Ting Y. L. Berti P. J. (2011). Lyme disease enolpyruvyl-UDP-GlcNAc synthase: fosfomycin-resistant MurA from *Borrelia burgdorferi*, a fosfomycin-sensitive mutant, and the catalytic role of the active site Asp. *Biochemistry* 12 2205–2212. 10.1021/bi1017842 21294548

[B15] Lee S. Y. Park Y. J. Yu J. K. Jung S. Kim Y. Jeong S. H. (2012). Prevalence of acquired fosfomycin resistance among extended-spectrum b-lactamase-producing *Escherichia coli* and *Klebsiella pneumoniae* clinical isolates in Korea and IS26-composite transposon surrounding *fosA3*. *J. Antimicrob. Chemother.* 67 2843–2847. 10.1093/jac/dks319 22893681

[B16] Michalopoulos A. S. Livaditis I. G. Gougoutas V. (2011). The revival of fosfomycin. *Int. J. Infect. Dis.* 15 e732–e739. 10.1016/j.ijid.2011.07.007 21945848

[B17] Sultan A. Rizvi M. Khan F. Sami H. Shukla I. Khan H. M. (2015). Increasing antimicrobial resistance among uropathogens, Is fosfomycin the answer? *Urol. Annal.* 7 26–30. 10.4103/0974-7796.148585 PMC4310112 25657539

[B18] Takahata S. Ida T. Hiraishi T. Sakakibara S. (2010). Molecular mechanisms of fosfomycin resistance in clinical isolates of *Escherichia coli*. *Int. J. Antimicrob. Agents* 2010 333–337. 10.1016/j.ijantimicag.2009.11.011 20071153

