# Supplementary material for: Prevalence of Fosfomycin Resistance and Mutations in murA, glpT, and uhpT in Methicillin-Resistant Staphylococcus aureus Strains Isolated from Blood and Cerebrospinal Fluid Samples
Source: Front Microbiol. 2016 Jan 11;6:1544. doi: 10.3389/fmicb.2015.01544 (PMC4707275; doi:10.3389/fmicb.2015.01544)
Supplement: Supplementary file 1 [file Table_1.DOC]

Supplementary Material

# Prevalence of fosfomycin resistance and mutations in *murA*, *glpT,* and *uhpT* in methicillin-resistant *Staphylococcus aureus* strains isolated from blood and cerebrospinal fluid samples

Zhuyingjie Fu^1, 2^, Ying Ma^1,2^, Chunhui Chen^1,2^, Yan Guo^1,2^, Fupin Hu^1,2^, Yang Liu^3^*, Xiaogang Xu^1^* and Minggui Wang^1,2^

*** Correspondence:** Xiaogang Xu and Yang Liu: [xuxiaogang@fudan.edu.cn](mailto:xuxiaogang@fudan.edu.cn); [liuyang@fudan.edu.cn](mailto:liuyang@fudan.edu.cn)

## Supplementary Tables

**Table S1. Fosfomycin MIC, *fosB* detection, mutations in *murA*, *glpT* and *uhpT* in 96 MRSA isolates**

| Number | Source | Year | Fosfomycin MIC(mg/L) | *fosB* | Mutation type  in *murA* | Mutation type  in *glpT* | Mutation type  in *uhpT* | MLST |
| --- | --- | --- | --- | --- | --- | --- | --- | --- |
| 2 | blood | 2004 | >1024 | - |  | TypeA*_glpT_* | TypeB*_uhpT_* | ST5 |
| 5 | blood | 2004 | >1024 | - | TypeII*_murA_* | TypeD*_glpT_* | TypeH*_uhpT_* | ST239 |
| 6 | blood | 2004 | >1024 | + |  | TypeA*_glpT_* | TypeB*_uhpT_* | ST5 |
| 8 | blood | 2004 | >1024 | - |  |  | TypeB*_uhpT_* | ST5 |
| 9 | blood | 2004 | >1024 | + |  |  | TypeA*_uhpT_* | ST5 |
| 14 | blood | 2005 | >1024 | - | TypeII*_murA_* | TypeA*_glpT_* | TypeB*_uhpT_* | ST5 |
| 16 | blood | 2005 | >1024 | + |  |  | TypeA*_uhpT_* | ST5 |
| 17 | blood | 2005 | >1024 | - | TypeII*_murA_* | TypeE*_glpT_* | TypeC*_uhpT_* | ST239 |
| 21 | blood | 2006 | >1024 | - |  |  | TypeB*_uhpT_* | ST5 |
| 24 | blood | 2006 | >1024 | - |  | TypeA*_glpT_* | TypeB*_uhpT_* | ST5 |
| 25 | blood | 2006 | >1024 | - |  | TypeA*_glpT_* | TypeB*_uhpT_* | ST5 |
| 26 | blood | 2006 | >1024 | - |  | TypeA*_glpT_* | TypeB*_uhpT_* | ST5 |
| 29 | blood | 2007 | >1024 | - |  |  | TypeB*_uhpT_* | ST5 |
| 37 | blood | 2008 | >1024 | - |  | TypeA*_glpT_* | TypeB*_uhpT_* | ST5 |
| 38 | blood | 2008 | >1024 | - |  | TypeA*_glpT_* | TypeB*_uhpT_* | ST5 |
| 44 | blood | 2008 | >1024 | - | TypeII*_murA_* |  | TypeD*_uhpT_* | ST239 |
| 45 | blood | 2008 | >1024 | - |  | TypeA*_glpT_* | TypeB*_uhpT_* | ST5 |
| 47 | blood | 2008 | >1024 | - |  | TypeA*_glpT_* | TypeB*_uhpT_* | ST5 |
| 49 | blood | 2008 | >1024 | + |  |  | TypeA*_uhpT_* | ST5 |
| 51 | blood | 2009 | >1024 | - |  | TypeA*_glpT_* | TypeB*_uhpT_* | ST5 |
| 56 | blood | 2010 | >1024 | - |  | TypeA*_glpT_* | TypeB*_uhpT_* | ST5 |
| 57 | blood | 2010 | >1024 | + |  | TypeA*_glpT_* | TypeA*_uhpT_* | ST764 |
| 58 | blood | 2010 | >1024 | - |  |  | TypeB*_uhpT_* | ST5 |
| 59 | blood | 2011 | >1024 | + |  | TypeA*_glpT_* |  | ST2590 |
| 62 | blood | 2011 | >1024 | - |  | TypeA*_glpT_* | TypeB*_uhpT_* | ST5 |
| 63 | blood | 2011 | >1024 | - |  | TypeA*_glpT_* | TypeB*_uhpT_* | ST5 |
| 64 | blood | 2011 | >1024 | - |  | TypeA*_glpT_* | TypeB*_uhpT_* | ST5 |
| 65 | blood | 2011 | >1024 | - |  | TypeA*_glpT_* | TypeB*_uhpT_* | ST5 |
| 66 | blood | 2011 | >1024 | - |  | TypeA*_glpT_* |  | ST5 |
| 68 | CSF* | 2011 | >1024 | - |  | TypeA*_glpT_* | TypeB*_uhpT_* | ST5 |
| 71 | blood | 2011 | >1024 | - |  | TypeB*_glpT_* | TypeB*_uhpT_* | ST5 |
| 72 | blood | 2011 | >1024 | - |  | TypeA*_glpT_* | TypeB*_uhpT_* | ST5 |
| 73 | blood | 2011 | >1024 | - |  | TypeA*_glpT_* | TypeB*_uhpT_* | ST5 |
| 74 | blood | 2011 | >1024 | - |  | TypeA*_glpT_* | TypeB*_uhpT_* | ST5 |
| 75 | CSF* | 2011 | >1024 | - |  | TypeA*_glpT_* | TypeB*_uhpT_* | ST5 |
| 76 | blood | 2012 | >1024 | - |  | TypeA*_glpT_* |  | ST5 |
| 80 | CSF* | 2012 | >1024 | + |  | TypeB*_glpT_* | TypeF*_uhpT_* | ST5 |
| 81 | blood | 2012 | >1024 | - |  | TypeA*_glpT_* | TypeB*_uhpT_* | ST5 |
| 82 | blood | 2012 | >1024 | - |  | TypeA*_glpT_* | TypeB*_uhpT_* | ST5 |
| 84 | blood | 2012 | >1024 | - |  | TypeA*_glpT_* | TypeB*_uhpT_* | ST5 |
| 88 | blood | 2012 | >1024 | - |  | TypeA*_glpT_* | TypeB*_uhpT_* | ST5 |
| 3 | CSF* | 2004 | 1024 | - |  | TypeB*_glpT_* | TypeC*_uhpT_* | ST5 |
| 7 | blood | 2004 | 1024 | - |  | TypeB*_glpT_* | TypeC*_uhpT_* | ST5 |
| 11 | blood | 2005 | 1024 | - |  | TypeB*_glpT_* | TypeC*_uhpT_* | ST5 |
| 15 | blood | 2005 | 1024 | - | TypeA*_murA_* | TypeB*_glpT_* | TypeC*_uhpT_* | ST5 |
| 19 | blood | 2006 | 1024 | - | TypeII*_murA_* | TypeE*_glpT_* | TypeG*_uhpT_* | ST239 |
| 41 | blood | 2008 | 1024 | - |  |  | TypeC*_uhpT_* | ST5 |
| 55 | blood | 2009 | 1024 | - |  | TypeB*_glpT_* | TypeC*_uhpT_* | ST5 |
| 67 | blood | 2011 | 1024 | - | TypeII*_murA_* | TypeA*_glpT_* | TypeE*_uhpT_* | ST239 |
| 69 | blood | 2011 | 1024 | - |  | TypeA*_glpT_* | TypeB*_uhpT_* | ST239 |
| 78 | CSF* | 2012 | 512 | + |  | TypeC*_glpT_* |  | ST5 |
| 94 | blood | 2014 | 512 | - |  |  | TypeA*_uhpT_* | ST764 |
| 97 | CSF* | 2014 | 512 | - | TypeII*_murA_* |  | TypeA*_uhpT_* | ST764 |
| 60 | blood | 2011 | 256 | - | TypeII*_murA_* |  |  | ST239 |
| 96 | blood | 2014 | 256 | - |  |  | TypeA*_uhpT_* | ST764 |
| 20 | blood | 2006 | 128 | + | TypeII*_murA_* |  |  | ST239 |
| 22 | CSF* | 2006 | 128 | - | TypeII*_murA_* |  |  | ST239 |
| 27 | CSF* | 2007 | 128 | - | TypeII*_murA_* | TypeA*_glpT_* |  | ST239 |
| 28 | CSF* | 2007 | 128 | - | TypeII*_murA_* |  |  | ST239 |
| 30 | CSF | 2007 | 128 | - | TypeII*_murA_* | TypeA*_glpT_* |  | ST239 |
| 61 | blood | 2011 | 128 | - |  |  | TypeB*_uhpT_* | ST5 |
| 91 | CSF* | 2013 | 128 | - |  |  | TypeA*_uhpT_* | ST239 |
| 18 | blood | 2005 | 64 | - | TypeII*_murA_* |  |  | ST239 |
| 39 | blood | 2008 | 64 | - | TypeII*_murA_* |  |  | ST239 |
| 40 | blood | 2008 | 64 | - | TypeII*_murA_* |  |  | ST239 |
| 70 | blood | 2011 | 64 | - |  | TypeB*_glpT_* |  | ST5 |
| 85 | blood | 2012 | 64 | - |  | TypeB*_glpT_* |  | ST5 |
| 1 | blood | 2004 | 32 | - |  |  |  | ST239 |
| 53 | blood | 2009 | 32 | - |  |  |  | ST863 |
| 98 | blood | 2014 | 32 | - | TypeII*_murA_* |  |  | ST239 |
| 36 | blood | 2007 | 16 | - |  |  |  | ST239 |
| 4 | blood | 2004 | 8 | - | TypeII*_murA_* |  |  | ST239 |
| 12 | blood | 2005 | 8 | - |  |  |  | ST239 |
| 23 | blood | 2006 | 8 | - | TypeII*_murA_* |  |  | ST239 |
| 34 | blood | 2007 | 8 | - | TypeII*_murA_* |  |  | ST239 |
| 35 | blood | 2007 | 8 | - | TypeII*_murA_* |  |  | ST239 |
| 13 | blood | 2005 | 4 | - |  |  |  | ST239 |
| 42 | blood | 2008 | 4 | - | TypeII*_murA_* |  |  | ST239 |
| 54 | blood | 2009 | 4 | - | TypeII*_murA_* |  |  | ST239 |
| 33 | blood | 2007 | 2 | - | TypeII*_murA_* |  |  | ST239 |
| 43 | blood | 2008 | 2 | - | TypeII*_murA_* |  |  | ST239 |
| 46 | blood | 2008 | 2 | - |  |  |  | ST239 |
| 48 | blood | 2008 | 2 | - | TypeI*_murA_*, TypeII*_murA_* |  |  | ST239 |
| 50 | blood | 2009 | 2 | - | TypeII*_murA_* |  |  | ST239 |
| 52 | blood | 2009 | 2 | - | TypeII*_murA_*, TypeV*_murA_* |  |  | ST239 |
| 79 | CSF* | 2012 | 2 | - | TypeIII*_murA_*, TypeIV*_murA_* | TypeIII*_glpT_*, TypeIV*_glpT_* |  | ST30 |
| 87 | blood | 2012 | 2 | - | TypeIV*_murA_*, TypeVI*_murA_* | TypeII*_glpT_*, TypeIII*_glpT_* |  | ST121 |
| 32 | blood | 2007 | 1 | - |  |  |  | ST239 |
| 77 | blood | 2012 | 1 | - | TypeIV*_murA_*, TypeVI*_murA_* | TypeIII*_glpT_* |  | ST59 |
| 83 | blood | 2012 | 1 | - | TypeIV*_murA_*, TypeVI*_murA_* | TypeIII*_glpT_* |  | ST59 |
| 86 | blood | 2012 | 1 | - | TypeIV*_murA_*, TypeVI*_murA_* | TypeIII*_glpT_* |  | ST59 |
| 89 | blood | 2013 | 1 | - |  |  |  | ST6 |
| 90 | blood | 2013 | 1 | - | TypeII*_murA_* |  |  | ST239 |
| 92 | blood | 2013 | 1 | - | TypeIII*_murA_*, TypeIV*_murA_* | TypeI*_glpT_*, TypeIII*_glpT_* |  | ST398 |
| 93 | CSF* | 2014 | 1 | - |  |  |  | ST88 |
| 95 | blood | 2014 | 1 | - | TypeIV*_murA_*, TypeVI*_murA_* | TypeIII*_glpT_*, TypeV*_glpT_* |  | ST59 |

*CSF: cerebrospinal fluid.
