# Supplementary material for: Prevalence of Fosfomycin Resistance and Mutations in murA, glpT, and uhpT in Methicillin-Resistant Staphylococcus aureus Strains Isolated from Blood and Cerebrospinal Fluid Samples
Source: Front Microbiol. 2016 Jan 11;6:1544. doi: 10.3389/fmicb.2015.01544 (PMC4707275; doi:10.3389/fmicb.2015.01544)
Supplement: Supplementary file 2 [file Table_2.DOC]

**Table S2. Characteristics of MRSA isolates contained *glpT* mutations**

| Types of mutation | Strain No. | Mutations in *glpT^*^* | Fosfomycin MIC range(mg/L) | *fosB* gene |
| --- | --- | --- | --- | --- |
| TypeA *_glpT_* | 30 | Deletion of 8bp from 225T to 232A (Truncated to 74 aa) | 128 ~ >1024 | Negative |
| TypeB *_glpT_* | 8 | G1064A (Truncated to 354 aa) | 64 ~ >1024 | Negative |
| TypeD *_glpT_* | 1 | Insertion of 392T (Truncated to 148 aa) | >1024 | Negative |
| TypeE *_glpT_* | 2 | T409C (Trp 137 Arg) | 1024 ~ >1024 | Negative |
| TypeI*_glpT_* | 1 | T7A (Phe 3 Ile) | 1 | Negative |
| TypeII*_glpT_* | 1 | C79T (Leu 27 Phe) | 2 | Negative |
| TypeIII*_glpT_* | 7 | C299T (Ala 100 Val) | 1 ~ 2 | Negative |
| TypeIV*_glpT_* | 1 | G637A (Val 213 Ile) | 2 | Negative |
| TypeV*_glpT_* | 1 | G1055A (Gly 352 Asp) | 1 | Negative |
| TypeA *_glpT_* | 3 | Deletion of 8bp from 225T to 232A (Truncated to 74 aa) | >1024 | Positive |
| TypeB *_glpT_* | 1 | G1064A (Truncated to 354 aa) | >1024 | Positive |
| TypeC *_glpT_* | 1 | deletion of 248G (Truncated to 84 aa) | 512 | Positive |

*Amino acid substitutions or sequence variations are shown in brackets.
