# Supplementary material for: Prevalence of Fosfomycin Resistance and Mutations in murA, glpT, and uhpT in Methicillin-Resistant Staphylococcus aureus Strains Isolated from Blood and Cerebrospinal Fluid Samples
Source: Front Microbiol. 2016 Jan 11;6:1544. doi: 10.3389/fmicb.2015.01544 (PMC4707275; doi:10.3389/fmicb.2015.01544)
Supplement: Supplementary file 3 [file Table_3.DOC]

**Table S3. Characteristics of MRSA isolates contained *uhpT* mutations**

| Types of mutation | Strain No. | Mutations in *uhpT^*^* | Fosfomycin MIC range(mg/L) | *fosB* gene |
| --- | --- | --- | --- | --- |
| TypeA*_uhpT_* | 4 | Deletion of 27T (Truncated to 32 aa) | 128 ~ 512 | Negative |
| TypeB*_uhpT_* | 31 | Insertion of 904T (Truncated to 301 aa) | 128 ~ >1024 | Negative |
| TypeC*_uhpT_* | 7 | G1073T (Gly 358 Val) | 1024 ~ >1024 | Negative |
| TypeD*_uhpT_* | 1 | G335A (Gly 112 Glu) | >1024 | Negative |
| TypeE*_uhpT_* | 1 | Deletion of 12bp from 431A to 442T (Truncated to 455 aa) | 1024 | Negative |
| TypeG*_uhpT_* | 1 | C942A (Truncated to 313 aa) | 1024 | Negative |
| TypeH*_uhpT_* | 1 | T1273C (Trp 425 Arg) | >1024 | Negative |
| TypeA*_uhpT_* | 4 | Deletion of 27T (Truncated to 32 aa) | >1024 | Positive |
| TypeB*_uhpT_* | 1 | Insertion of 904T (Truncated to 301 aa) | >1024 | Positive |
| TypeF*_uhpT_* | 1 | G683A (Truncated to 227 aa) | >1024 | Positive |

*Amino acid substitutions or sequence variations are shown in brackets.
